# Vaginal Microbiota Evaluation and Lactobacilli Quantification by qPCR in Pregnant and Non-pregnant Women: A Pilot Study

**DOI:** 10.3389/fcimb.2020.00303

**Published:** 2020-06-19

**Authors:** David Pacha-Herrera, Gabriela Vasco, Cecilia Cruz-Betancourt, Juan Miguel Galarza, Verónica Barragán, António Machado

**Affiliations:** ^1^Laboratorio de Bacteriología, Instituto de Microbiología, Colegio de Ciencias Biológicas y Ambientales, Universidad San Francisco de Quito, Quito, Ecuador; ^2^Cátedra de Microbiología de la Escuela de Medicina, Facultad de Ciencias Médicas, Universidad Central del Ecuador, Quito, Ecuador; ^3^Laboratorio de Biología Molecular, Unidad Técnica de Genética y Molecular, Hospital de Especialidades Carlos Andrade Marín del IESS, Quito, Ecuador

**Keywords:** vaginal microbiota, vaginal infection, bacterial vaginosis, aerobic vaginitis, pregnant, opportunistic pathogen, *Lactobacillus* spp.

## Abstract

Pregnancy outcomes and women's health are directly affected by vaginal microbiota. This microbiota consists of a dynamic ecosystem of various microbes in different ratios, which in healthy conditions protect the vaginal epithelium from infections. However, cases of vaginal infection are regularly diagnosed in women of reproductive age, contributing to more severe outcomes. Therefore, our main goal was to determine the prevalence of bacterial vaginosis (BV), aerobic vaginitis (AV), and vulvovaginal candidiasis (VVC) among Ecuadorian pregnant and non-pregnant women. A cross-sectional study was conducted among 217 women between 13 and 40 years old seeking primary healthcare in Carlos Andrade Marin Hospital (HCAM), Gynecological-Obstetric Hospital Isidro Ayora (HGOIA) and Center for Teaching Health Cipriana Dueñas during October 2018 to February 2019. The classical characterization of the vaginal microbiota was performed through microscopy by the Nugent criteria to evaluate the presence of BV, healthy and intermediate microbiota, by the criteria of Donders to determine the presence of AV and by the Marot-Leblond criteria to diagnose VVC. DNA extraction from vaginal samples and Polymerase Chain Reaction (PCR) analysis was performed to characterize the presence of *Gardnerella* spp., *Mobiluncus mulieris, Escherichia coli, Enterococcus* spp., and *Lactobacillus* spp. Finally, quantification of the lactobacilli was performed by quantitative real-time PCR (qPCR) for samples from women with normal vaginal microbiota and women with AV. Our results showed 52% of women with healthy microbiota, 7% with intermediate microbiota, and 41% with vaginal dysbiosis, comprising 27% with AV, 8% with BV and 4% with VVC and 2% with co-infections or co-dysbiosis. Additionally, a higher amount of lactobacilli were found in pregnant women when compared to non-pregnant women, while AV cases were characterized by a significant drop of *Lactobacillus* spp., more precisely, between 1E3 and 1E5 colony forming units (CFU)/ml. Finally, women with normal vaginal microbiota showed an average load of lactobacilli between 1E6 and 1E7 CFU/ml. This pilot study showed no statistically significant differences between pregnant and non-pregnant women, pointing to the possibility to use lactobacilli quantification for the prevention of future vaginal infections.

## Introduction

The normal vaginal microbiota plays a crucial role for the health of pregnant and non-pregnant women (Vaneechoutte, 2017b), preventing several urogenital diseases (Ling et al., 2013), including bacterial vaginosis (BV) (Dai et al., 2010; Ling et al., 2010, 2013; Gondo et al., 2011; Van De Wijgert et al., 2014), aerobic vaginitis (AV) (Donders et al., 2005, 2011; Fan et al., 2013; Jahic et al., 2013; Tansarli et al., 2013), urinary tract infections (UTI) (Cauci et al., 2002; Zhou et al., 2004; Borges et al., 2014), yeast vaginitis (Ringdahl, 2006; Dai et al., 2010; Xu et al., 2010), and sexually transmitted diseases (such as HIV) (Bolton et al., 2008; Srinivasan and Fredricks, 2008; Petrova et al., 2013; Van De Wijgert et al., 2014). In the context of this study, it is also important to mention that women and teenagers in Ecuador have a wide range of health care needs, in particular, related to sexual and reproductive health (Svanemyr et al., 2017). In Ecuador, a major concern is the high rate of adolescent pregnancy, i.e., pregnancy between ages 10 to 19. Several studies worldwide also demonstrated a higher risk of acquiring HIV, herpes simplex virus type 2 and other sexually transmitted infections in non-pregnant women with vaginal infections or intermediate vaginal microbiota (Li et al., 2012; Petrova et al., 2013; Datcu et al., 2014; Van De Wijgert et al., 2014). Thus, lactic acid-producing bacteria (such as *Lactobacillus* spp.) metabolize glycogen, increasing lactic acid and a normal acidic vaginal pH of 3.8–4.4 (Farage et al., 2010; Borges et al., 2014; Mendling, 2016; Vaneechoutte, 2017b).

The vaginal microbial community is a variable econiche that fluctuates between normal and dysbiotic microbiota (Vaneechoutte, 2017b), which could be influenced by several intrinsic and extrinsic factors (Mendling, 2016) and eventually leading to an increment of both aerobic and anaerobic microorganisms (Larsen and Monif, 2001; Ling et al., 2010; Fredricks, 2011; Ravel et al., 2011). However, the most predominant genus in a healthy vaginal microbiota is *Lactobacillus* (Borges et al., 2014; Vaneechoutte, 2017b). *Lactobacillus* genus is known to inhibit the adhesion and proliferation of opportunistic and primary pathogens (Bolton et al., 2008). The mechanisms by which vaginal lactobacilli provide colonization resistance is generally considered to be through production of several antimicrobial compounds such as hydrogen peroxide, lactic acid and/or bacteriocins (Aroutcheva et al., 2001; Alpay et al., 2003; Vaneechoutte, 2017b; Collins et al., 2018), as well as acting as biosurfactant on the vaginal epithelium (Boris and Barbés, 2000; Borges et al., 2014).

Although several species of *Lactobacillus* were already identified in vaginal microbiota, the most predominant species found in normal vaginal microbiota are *L. crispatus, L. gasseri, L. jensenii*, and *L. iners* (Farage et al., 2010; Borges et al., 2014; Oliveira et al., 2018). Also, other species could be detected in low amount among healthy vaginal microbiota such as *Atopobium, Enterobacter, Escherichia, Gardnerella, Mobiluncus, Prevotella, Staphylococcus, Shigella* (Hernández-Rodríguez et al., 2011; Gajer et al., 2012; Romero et al., 2014; Oliveira et al., 2018). These species can also behave as opportunistic pathogens (Gajer et al., 2012; Vaneechoutte, 2017a). Several factors can induce disruptions of the healthy microbiota equilibrium, establishing a microbial dysbiosis and, thus, future vaginal infections (Gajer et al., 2012; Johnson and Versalovic, 2012; Petrova et al., 2013; Vaneechoutte, 2017b).

According to previous studies, bacterial vaginosis (BV) is the most common vaginal dysbiosis among women of reproductive age (Cristiano et al., 1996; Nelson et al., 2009; Dai et al., 2010; Gondo et al., 2011), being characterized by lactobacilli replacement by anaerobes (Donders et al., 2011). *Gardnerella* spp., *Atopobium vaginae, Bacteroides* spp. and *Mobiluncus* spp. are the main pathogenic anaerobes associated with BV (Mendling, 2016), which is usually diagnosed by Nugent criteria (Nugent et al., 1991) or the Amsel criteria (Van De Wijgert et al., 2014). Besides BV, a condition designated aerobic vaginitis (AV) has also been recognized, characterized by the presence of aerobic bacteria in detriment of lactobacilli and by inflammation diagnosed a yellow-green discharge (Donders, 2007; Mendling, 2016). This vaginal infection is usually dominated by *Streptococcus* sp., *Enterococcus* sp., and/or Gram-negative bacteria of enteric origin (mainly, *Escherichia coli*). Finally, vulvovaginal candidiasis (VVC) is the most prevalent cause of vaginal infection by fungi, with at least 75% of healthy women suffering one episode of VVC during lifetime (Ringdahl, 2006) and whereby *Candida albicans* is the most important species (Marot-Leblond et al., 2009).

Our main goal of the present study was to evaluate the presence of vaginal infection among Ecuadorian women by classical and standard microbiological techniques or criteria (Fredricks, 2011) and to determine the dominance of different types of vaginal infection among pregnant and non-pregnant women. Also, the present study aimed to detect the presence of specific opportunistic pathogens (*E. coli, Enterococcus faecalis, Gardnerella* spp., and *Mobiluncus mulieris*) by Polymerase Chain Reaction (PCR) and quantified the number of lactobacilli through quantitative real-time PCR (qPCR). The analysis of the normal amount of lactobacilli in pregnant and non-pregnant women might enable to determine the lactobacilli threshold associated with the establishment of vaginal infection.

## Materials and Methods

### Study Population, Design, and Subject Selection

The study was conducted in the Microbiology Institute at USFQ in collaboration with Hospital Carlos Andrade Marín (HCAM) and Universidad Central del Ecuador (UCE) from October 2018 to February 2019. The research team recruited 217 Ecuadorian female volunteers of Hispanic ethnicity but in reproductive age (13 and 40 years old), of which 111 were pregnant. Applicants were excluded from the study if they reported antimicrobial treatment in the last 3 months or any evidence of bleeding, and also if they had sexual intercourse within the previous 48 h. Also, a questionnaire was taken regarding demographic characteristics, sexual and health behavior of each patient, and each enrolled woman provided a usable vulvovaginal swab sample.

### Ethics Statement

This study was approved by the Ethics Committee of Universidad San Francisco de Quito (USFQ) and the Ministry of Health of Ecuador (Protocol code: 2016-140M by MSP-SDM-10-2013-2019-O review board). The female participants were recruited to our study set, after having read and signed the informed consent or, in the case of underaged participants, from their parents or legal representatives.

### Sample Collection

Samples were taken by a gynecologist using a sterile disposable vaginal speculum. The lateral vaginal walls were swabbed with a sterile swab to collect the cervical fluid, to prepare a smear on a microscope slide. Briefly, each vaginal smear was obtained by rolling the previous swab onto a glass slide, then heat-fixed and Gram-stained by using safranin as the counterstain. Following the Gram smear procedure, the swab was placed in 1 ml of phosphate buffer saline (PBS) and vortexed vigorously for ~3 min. The remaining vaginal material was collected by centrifugation at 16,000 g for 5 min. The obtained pellet was suspended into an aliquot of 1 ml of saline (0.9% NaCl) which was used for culture of *Candida* spp. in different media (see section Culture of Candida spp.) and for wet mount microscopy for a better diagnosis of AV and VVC (see section Microbiological Classification of Vaginal Infections).

A second sample was taken by a cervical brush (Rovers Cervex Brush®) through endo and exo-cervical brushing, placed immediately in Cobas® Preservative Fluid, stored at 4°C until processing in the clinical laboratory of HCAM, and used for DNA extraction (see section DNA Extraction of Vaginal Swabs). Each sample was further used to culture of *Candida* spp.

### Culture of *Candida* spp.

*Candida* spp. was cultured on different media from the saline aliquot (see section Sample Collection). Briefly, 100 μl of saline solution was plated onto Petri dishes containing 5% human blood agar (HBA), chocolate agar (heated human blood agar) or Sabouraud dextrose agar (SDA). The plates were incubated at 37°C for 48 h, under aerobic conditions, and colonies were analyzed and identified by gram staining, biochemical properties (catalase, oxidase, and hemolysis) and PCR (data not shown).

### Microbiological Classification of Vaginal Infections

The Gram-stained vaginal smears were classified according to Nugent criteria for bacterial vaginosis (BV) (Nugent et al., 1991), the criteria of Donders et al. for aerobic vaginitis (AV) (Donders, 1999) and those of Marot-Leblond et al. for vulvovaginal candidiasis (VVC) (Marot-Leblond et al., 2009). The evaluation of several cell types present in each smear was performed for 10 to 15 microscopic fields under oil immersion at 1000 X magnification (Donders, 2007).

After an initial evaluation of the Gram-stained smears by the Nugent criteria, all samples were evaluated by means of phase-contrast microscopy (X400 magnification) of wet smears, according to Schröders classification (Donders et al., 2005) and the Marot-Leblond et al. (2009) criteria (see Table 1). The absence of *Lactobacillus* spp., presence of cocci or coarse bacilli in high numbers, presence of parabasal epithelial cells representing >10% of the epithelial cells, and/or presence of leucocytes were considered as indicative for AV (Donders et al., 2005). In addition, aggravated AV diagnosis was defined as the most extreme form of aerobic vaginitis under Donders evaluation from Schröders classification (Donders et al., 2005), where AV samples showed lactobacilli severely depressed or absent because of overgrowth of other bacteria (Cocci or chains), more than 10 leukocytes per epithelial cell present in the samples and more than 50% of the leukocytes had a toxic appearance. It is important to mention that leukocytes were also evaluated on their granular appearance due to abundant lysozyme activity (“toxic leukocytes”) (Donders et al., 2005). Finally, VVC was assessed accordingly to Marot-Leblond and colleagues through at least one of the following criteria: positive Gram-stain preparation with budding yeasts in high numbers (five or more) in more than two microscopic fields, pseudohyphae, and/or hyphal forms in wet smears observation; and positive culture in Chocolate agar, HBA and/or SDA, along with negative microscopic examination results associated with eventual symptoms (thick, white vaginal discharge with no odor, vulvar and vaginal pruritus, burning, or dyspareunia) or clinical history (previous infection) obtained from the medical survey with the professional gynecologist. Absence of *Candida* cells in more than two microscopic fields and/or a low number of *Candida* spp. result on wet smears observation and culture growth was considered as normal *Candida* colonization rather than VVC (Marot-Leblond et al., 2009).

**Table 1:** Parameters used for the diagnosis of vaginal infections.

**Infection**	**Symptoms**	**Discharge**	**Odor**	**Diagnosis**	**References**
Vulvovaginal candidiasis	Pruritus	Thick, white to yellow	Absent	Microscopic examination (Gram-stained smears and Wet mount preps), medical survey and growth culture	Carr et al., 1998; Marot-Leblond et al., 2009
Aerobic vaginitis	Inflammation	Yellow	Foul, rotten	Microscopic examination (Gram-stained smears and Wet mount preps) and medical survey	Donders et al., 2002; Donders et al., 2005
Bacterial vaginosis	Irritation, 50% asymptomatic	Thin, white to gray, homogeneous	Fishy	Microscopic examination (Gram-stained smears and Wet mount preps) and medical survey	Carr et al., 1998; Nugent et al., 1991

### DNA Extraction of Vaginal Swabs

The Cobas® 4800 system (Roche Molecular Systems Inc., Pleasanton, CA) was used to extract the DNA of vaginal brushes, according to the manufacturer's instructions. DNA was quantified with a Nanovue spectrophotometer (GE Healthcare Life Science). DNA was eluted at 20 ng/μl with molecular grade water and stored at −20°C until the Polymerase Chain Reaction (PCR) analysis was performed. The quality of DNA was evaluated by measuring the concentration of phenolic compounds or the presence of salts (260/230) and protein contaminants (260/280).

### Identification of the Major Bacterial Species by PCR

From 217 vaginal samples previously diagnosed by classical criteria through microscopy analysis, 97 were selected for molecular characterization by PCR in a Bio-Rad Thermocycler (Bio-Rad, Hercules, CA). Samples with scores between 0 and 1 of Nugent criteria were selected as healthy microbiota, while samples with scores between 9 and 10 of Nugent criteria (BV) and diagnosed as representing aggravated AV (see section Microbiological Classification of Vaginal Infections) were used as dysbiotic microbiota. Thus, sixty samples with healthy microbiota (38 pregnant and 22 non-pregnant women), 23 samples with AV (14 pregnant and 9 non-pregnant women), and 14 samples with BV microbiota (6 pregnant and 8 non-pregnant women) were included. All samples were analyzed with a total of five primer pairs, targeting two anaerobes (*Gardnerella* species, and *M. mulieris*), two aerobes (*E. coli* and *E. faecalis*) and for the genus *Lactobacillus*. Single-template PCR assays were performed for each primer set. The sequence, amplicon size, target gene, and temperature of annealing for each primer pair are described in Table 2.

**Table 2:** PCR primers used in this study.

**Set**	**Name**	**Sequence (5^**′**^-3^**′**^)**	**Target**	**T (^**°**^C) of annealing**	**Size of fragment**	**Target gene**	**Specificity %**	**Validation**	**References**
1	Primer E1	ATCAAGTACAGTTAGTCTT	*Enterococcus faecalis*	54°C	941 bp	*ddl*	100.0%	Increase of the annealing temperature at 54°C	DTU National Food Institute, 2014
	Primer E2	ACGATTCAAAGCTAACTG							
2	adk F	ATTCTGCTTGGCGCTCCGGG	*Escherichia coli*	57°C	583 bp	*adk*	49.0% 98.0%	Increase of the annealing temperature at 57°C	Sepehri et al., 2009
	adk R	CCGTCAACTTTCGCGTATTT							
3	Gard154-Fw	CTCTTGGAAACGGGTGGTAA	*Gardnerella* spp.	60°C	301 bp	*16S* rRNA	100.0%	N/d	Henriques et al., 2012
	Gard154-Rv	TTGCTCCCAATCAAAAGCGGT							
4	LactoF	TGGAAACAGRTGCTAATACCG	*Lactobacillus* spp.	62°C	233 bp	*16S* rRNA	47.1% 66.7%	N/d	Henriques et al., 2012
	LactoR	GTCCATTGTGGAAGATTCCC							
5	Mobil-577F	GCTCGTAGGTGGTTCGTCGC	*Mobiluncus mulieris*	62°C	449 bp	*16S* rRNA	100.0%	N/d	Fredricks et al., 2007
	M.mulie-1026R	CCACACCATCTCTGGCATG							

*N/d – Non-determined*.

For PCR, a final volume of 20 μl was used according to the reference protocols (Galán et al., 2006; Fredricks et al., 2007; Sepehri et al., 2009; Henriques et al., 2012; DTU National Food Institute, 2014); which included 0.5 U of Go Taq® DNA Polymerase (Promega, Madison, WI), 1X of Green GoTaq® Flexi Buffer (Promega), 0.25 mM of MgCL_2_ (Promega), 200 μM of dNTP mix (Promega), 0.5 μM of each primer and target template DNA concentration of ~4 ng/μL, and the remaining volume with molecular grade H_2_O. The PCR thermal cycling consisted of initial denaturation at 94°C for 2 min; followed by 29 cycles of denaturation at 94°C for 30 s, annealing at each primer pair temperature (Table 2) for 30 s and extension at 72°C for 1 min, and final extension of 5 min at 72°C. The respective use of negative (without DNA sample and samples with other related bacteria) and positive (collection of identified strains of each species through DNA sequencing) controls were used in each PCR assay. These positive controls were provided by the Microbiology Institute at USFQ. All samples were randomly performed in duplicate or triplicate with different negative and positive controls.

After PCR amplification, a volume of 4 μL from each PCR product was visualized in 1.5% (w/w) agarose (Promega) gel electrophoresis using 0.1% ethidium bromide staining. The DNA analysis was performed under permit No. MAE-DNB-CM-2016-0046.

### Quantification of *Lactobacillus* sp. by Quantitative Real-Time PCR (qPCR)

To create positive controls and standard quantification solutions with a well-known *Lactobacillus* sp. concentration (CFU/mL), a sample of known concentration (also known as a calibrator) (e.g., number of CFU per mL) was obtained through a validated calibration curve (CFU/OD) (Begot et al., 1996). This calibrator was serially diluted tenfold and used to construct a standard curve for qPCR assays. Accordingly, *Lactobacillus gasseri* strain JCM1131 was cultured during 24 h in Mann Rogosa Sharp Agar at 37°C under microaerophilic conditions (Begot et al., 1996; Mytilinaios et al., 2012; Machado et al., 2013). The calibrator concentration was previously proved by media growth culture counting as previously described (Naghili et al., 2013). The DNA extraction was performed from the highest CFU/ml concentration, and serial dilutions from 1E9 to 1E0 CFU/mL were used as qPCR standards. The DNA extraction of this solution with the highest concentration was performed under the same procedure already described in section DNA Extraction of Vaginal Swabs. In each qPCR assay, two random controls were also used as blind samples in triplicate.

Each reaction was performed with GoTaq® Master Mix qPCR (Promega, Madison, WI, USA) in a final volume of 20 μl, 0.5 μM of each primer (LactoF-TGGAAACAGRTGCTAATACCG and LactoR-GTCCATTGTGGAAGATTCCC) and 2 μl of DNA template. Each qPCR assay was performed in a quantitative real-time PCR Thermocycler (Bio-Rad CA, USA) under the following conditions: initial denaturation at 94°C for 2 min followed by 40 cycles of denaturation at 94°C for 30 s, annealing at 62°C for 30 s, extension at 72°C for 1 min, and a final extension for 5 min at 72°C. Each qPCR assay was followed by this melt curve analysis, allowing amplicon validation and identification of false positives through its profile and the specific temperature of melting (Tm). Each sample was analyzed by triplicate, and qPCR assays were realized in different days. Negative target controls and no template controls were included in all plates.

Primers used for these qPCR assays were previously described to amplify *Lactobacillu*s spp. through classical PCR, but not for quantitative real-time PCR. Therefore assay metrics were determined by testing their performance across the limit of quantification (LoQ) and limit of detection (LoD), as well as linearity as previously described (Price et al., 2012). The optimized assay exhibited the LoQ and LoD to be 1E2 CFU per ml, while the range of linearity of the assay was from 1E9 to 1E3 CFU/mL. The load of lactobacilli in each sample was determined by running six or five standard dilutions (1E9-1E3 CFU/mL), both in duplicate or triplicate on each qPCR assay.

For the quantification of *Lactobacillus* spp., 83 samples were selected for qPCR analysis from the initial subset of 97 vaginal samples previously characterized by PCR assays to molecular characterization of the main bacteria (see section Identification of the Major Bacterial Species by PCR), i.e., 60 from healthy microbiota samples and 23 from AV samples.

### Statistical Analysis

Statistically significant differences in *Lactobacillus* spp. quantity among women with healthy and dysbiotic microbiota were evaluated using Kruskal Wallis one-way ANOVA and Mann Whitney tests. In addition, the same statistical analysis was carried out among pregnant and non-pregnant women. Finally, multivariable analysis was performed for sociodemographic and behavioral factors by using Minitab 2017 (Version 17, Minitab, State College, PA).

## Results

### Population Study

The sociodemographic characteristics for 217 women were included in the statistical analysis and presented in Table 3. Half of the women in the study were pregnant (51.2%) and approximately half (47.4%) were non-pregnant. They were between 21 and 30 years of age. Only 11 women (5.1%) identified themselves as White, Afro-Ecuadorian, or Indigenous women. So, the majority of the women in our study set (94.9%) were categorized as “Half-blood,” being of Hispanic ethnicity mixed with another background ethnicity (Caucasian, African, or Indigenous women). When performing an overall statistical analysis of age, the results do not show a significant relationship between age and the probability of having a specific diagnosis. Hence, there is no statistical evidence to determine that a woman's age is directly related to a specific vaginal disruption or having a healthy microbiota. From all sociodemographic factors analyzed, only the occupation category had a statistical significance over the diagnostic classification of vaginal infection with a *P*-value of 0.003 through the Chi-square test (see Table 3). Similarly, the use of contraceptive methods, having different sexual partners, vaginal douches, or cigarette smoking did not show any relation to the development of any vaginal infection type during the study (see Table 4).

**Table 3:** Sociodemographic among women in this study with healthy vaginal microbiota, intermediate vaginal microbiota, and vaginal infections (bacterial vaginosis, aerobic vaginitis, candidiasis, and co-infections).

	**Healthy microbiota *N* (%)**	**Intermediate microbiota *N* (%)**	**Candidiasis *N* (%)**	**Bacterial vaginosis *N* (%)**	**Aerobic vaginitis *N* (%)**	**Co-infections *N* (%)**	**Total *N***	***P* (X^**2**^)**
**Focus group**								
Non-pregnant	52 (49.1)	7 (6.6)	4 (3.8)	12 (11.3)	28 (26.4)	3 (2.8)	106	0.566 (3.9)
Pregnant	60 (54.1)	9 (8.1)	5 (4.5)	6 (5.4)	30 (27.0)	1 (0.9)	111	
**Age**								
≤ 20	38 (47.5)	4 (5.0)	5 (6.3)	10 (12.5)	21 (26.3)	2 (2.5)	80	0.799 (92.7)
21–25	26 (41.9)	6 (9.7)	1 (1.6)	4 (6.5)	24 (38.7)	1 (1.6)	62	
26–30	24 (58.5)	3 (7.3)	3 (7.3)	2 (4.9)	9 (21.9)	0 (0.0)	41	
31–40	24 (70.6)	3 (8.8)	0 (0.0)	2 (5.9)	4 (11.8)	1 (2.9)	34	
***Global Incidence***	112 (52.0)	16 (7.0)	9 (4.0)	18 (8.0)	58 (27.0)	4 (2.0)	217	0.308 (22.6)
**Ethnicity**								
Afro Ecuadorian	1 (25.0)	1 (25.0)	0 (0.0)	0 (0.0)	2 (50.0)	0 (0.0)	4	0.737 (11.2)
Half-blood	107 (51.9)	15 (7.3)	9 (4.4)	18 (8.7)	53 (25.7)	4 (1.9)	206	
Indigenous	0 (0.0)	0 (0.0)	0 (0.0)	0 (0.0)	2 (100.0)	0 (0.0)	2	
White	4 (80.0)	0 (0.0)	0 (0.0)	0 (0.0)	1 (20.0)	0 (0.0)	5	
**Occupation**								
Housewife	31 (46.3)	1 (1.5)	1 (1.5)	11 (16.4)	22 (32.8)	1 (1.5)	67	0.003 (26.7)
Student	39 (46.4)	6 (7.1)	5 (6.0)	5 (6.0)	26 (31.0)	3 (3.6)	84	
Employee	42 (63.6)	9 (13.6)	3 (4.5)	2 (3.0)	10 (15.2)	0 (0.0)	66	
**Civil Status**								
Married	24 (63.2)	3 (7.9)	0 (0.0)	2 (5.3)	8 (21.1)	1 (2.6)	38	0.245 (18.4)
Divorced	1 (50.0)	1 (50.0)	0 (0.0)	0 (0.0)	0 (0.0)	0 (0.0)	2	
Single	58 (49.6)	6 (5.1)	6 (5.1)	7 (6.0)	37 (31.6)	3 (2.6)	117	
Free Union	29 (48.3)	6 (10.0)	3 (5.0)	9 (15.0)	13 (21.7)	0 (0.0)	60	
**Education Level**								
None	2 (66.7)	1 (33.3)	0 (0.0)	0 (0.0)	0 (0.0)	0 (0.0)	3	0.916 (24.2)
Basic (High-school students)	13 (41.9)	2 (6.5)	0 (0.0)	4 (12.9)	10 (32.3)	2 (6.5)	31	
Bachelor (Undergraduate students)	52 (47.7)	10 (9.2)	7 (6.4)	9 (8.3)	30 (27.5)	1 (0.9)	109	
Superior (Bachelor graduates)	30 (55.6)	3 (5.6)	1 (1.9)	5 (9.3)	14 (25.9)	1 (1.9)	54	
Higher Degree Research (HDR) candidates (Master and Doctor's degree students)	15 (75.0)	0 (0.0)	1 (5.0)	0 (0.0)	4 (20.0)	0 (0.0)	20	

*N number of women who responded in the survey within each category; % assigned percentage for each classification within each category. P (X2) p-value through the Chi-square test show any relation among each sociodemographic factor and the possibility of having a vaginal disruption or healthy microbiota*.

**Table 4:** Behavioral variables among women in this study with healthy vaginal microbiota, intermediate vaginal microbiota, and vaginal infections (bacterial vaginosis, aerobic vaginitis, candidiasis, and co-infections).

	**Healthy microbiota *N* (%)**	**Intermediate microbiota *N* (%)**	**Candidiasis *N* (%)**	**Bacterial vaginosis *N* (%)**	**Aerobic vaginitis *N* (%)**	**Co-infections *N* (%)**	**Total *N***	***P* (X^**2**^)**
**Has sexual partner**								
No	16 (45.7)	3 (8.6)	3 (8.6)	2 (5.7)	10 (28.6)	1 (2.9)	35	0.707 (3.0)
Yes	96 (52.7)	13 (7.1)	6 (3.3)	16 (8.8)	48 (26.4)	3 (1.6)	182	
**Different sexual partners**								
No	54 (58.1)	4 (4.3)	4 (4.3)	7 (7.5)	24 (25.8)	0 (0.0)	93	0.205 (13.3)
Yes	52 (49.1)	10 (9.4)	5 (4.7)	7 (6.6)	29 (27.4)	3 (2.8)	106	
Do not answer	6 (33.3)	2 (11.1)	0 (0.0)	4 (22.2)	5 (27.8)	1 (5.6)	18	
**Uses birth control method**)								
No	45 (46.9)	1 (25.0)	6 (6.3)	7 (7.3)	29 (30.2)	1 (1.0)	96	0.877 (5.2)
Yes	65 (55.1)	15 (7.3)	3 (2.5)	11 (9.3)	28 (23.7)	3 (2.5)	118	
Do not answer	2 (66.7)	0 (0.0)	0 (0.0)	0 (0.0)	1 (33.3)	0 (0.0)	3	
**Smokes**								
No	105 (51.5)	14 (6.9)	8 (3.9)	18 (8.8)	55 (27.0)	4 (2.0)	204	0.683 (3.1)
Yes	7 (53.8)	2 (15.4)	1 (7.7)	0 (0.0)	3 (23.1)	0 (0.0)	13	
**Vaginal douching**								
No	31 (57.4)	3 (5.6)	1 (1.9)	6 (11.1)	12 (22.2)	1 (1.9)	54	0.881 (5.2)
Yes	79 (49.7)	12 (7.5)	8 (5.0)	12 (7.5)	45 (28.3)	3 (1.9)	159	
Do not answer	2 (50.0)	1 (25.0)	0 (0.0)	0 (0.0)	1 (25.0)	0 (0.0)	4	

*N number of women who responded in the survey within each category; % assigned percentage for each classification within each category. P (X2) P-value through the Chi-square test*.

### Diagnosis of Vaginal Infections

The vaginal samples were evaluated in the Microbiology Institute of USFQ, according to microbiological criteria of Nugent et al. (1991) to identify healthy microbiota, BV, and intermediate microbiota (Nugent et al., 1991); Schröders classification under criteria of Donders et al. (2005) to characterize AV (Donders et al., 2005), and the criteria of Marot-Leblond et al. (2009) to determine VVC (Marot-Leblond et al., 2009). As shown in Table 3, 112 (52.0%) vaginal samples were classified as healthy microbiota, 16 (7.0%) were identified as intermediate microbiota, and 89 (41.0%) were diagnosed as dysbiotic (41.0%), which includes single cases of BV, AV, and VVC but also co-infections. The presence of a unique type of vaginal infection was identified in 85 vaginal samples (39.2%), whereby AV was the most prevalent infection with 26.7% of the vaginal samples, followed by BV (8.3%) and 4.1% with VVC. Furthermore, four vaginal samples were diagnosed with co-infections (1.8%), more precisely two of them with AV and BV, one with AV and VVC, and one with BV and VVC. None of the co-infection samples was further evaluated during qPCR analysis.

#### Prevalence of Vaginal Infections Among Pregnant and Non-pregnant Women

Each focus group (pregnant and non-pregnant women) was analyzed to identify any relation between vaginal infection and pregnancy (see Table 5). Although pregnant and non-pregnant women have similar prevalence values in the healthy microbiota, most cases of BV and co-infection were found in non-pregnant women with 67% (12/18) and 75% (3/4) of the cases, respectively, as shown in Table 5. However, these differences were not significant.

**Table 5:** Contingency table of vaginal samples between Focus Group and the diagnosis of vaginal infections, healthy and intermediate vaginal microbiota.

		**Diagnostic**						
**Group**		**Aerobic vaginitis**	**Bacterial vaginosis**	**Candidiasis**	**Co-infection**	**Healthy**	**Intermediate**	**Total**
Non-Pregnant	Number	28	12	4	3	52	7	106
	(% within the column)	(48.3)	(66.7)	(44.4)	(75.0)	(46.4)	(43.8)	(48.8)
Pregnant	Number	30	6	5	1	60	9	111
	(% within the column)	(51.7)	(33.3)	(55.6)	(25.0)	(53.6)	(56.3)	(51.2)

*Number of women who responded in the survey within each category; % assigned percentage for each classification within each category. No statistically significant differences were found between pregnant and non-pregnant groups among vaginal infections, healthy and intermediate microbiota (P-value = 0.566[3.888]; see Table 3)*.

### Presence of Opportunistic Species and *Lactobacillus* spp. in Vaginal Microbiota

The presence of *G. vaginalis* and *M. mulieris* (as BV biomarkers), *E. coli* and *E. faecalis* (as AV biomarkers), and *Lactobacillus* spp. (as healthy biomarker) were analyzed by PCR assays from the selected 97 samples (see section Identification of the Major Bacterial Species by PCR). As previously mentioned, almost a half of population set was chosen by classical criteria through microscopy analysis (data not shown), more exactly, healthy microbiota samples with 0–1 and BV samples with 9–10 according to Nugent criteria (Nugent et al., 1991), and the most aggravated AV samples (Donders, 1999).

The results still evidenced the presence of *Lactobacillus* spp. in both types of vaginal dysbiosis, although their presence decreased to 21% in BV (*P* = 0.006) and 13% in AV (*P* = 0.019) when compared to healthy microbiota samples (see Figure S1). Regarding the presence of *Gardnerella* species, it was present less frequently in healthy microbiota (37%) while in BV and AV prevalence was 71% (*P* = 0.001) and 78% (*P* = 0.033), respectively. On the other hand, *M. mulieri*s and *E. coli* were found in BV at 79% and 36%, respectively; while being detected in low frequency in healthy microbiota and AV cases, as shown in Figure S1. The presence of *M. mulieris* was low in frequency on AV and normal microbiota, when compared to BV cases. However, presence of *M. mulieris* was statistically different among healthy microbiota against BV (*P* < 0.001) and AV cases (*P* = 0.002), being less recurrent in healthy samples. Finally, *E. coli* did not show statistical differences among healthy microbiota and BV cases (*P* = 0.062). Also, it is important to mention that *E. faecalis* was found to be absent in the population set of the present study.

**Figure 1:**
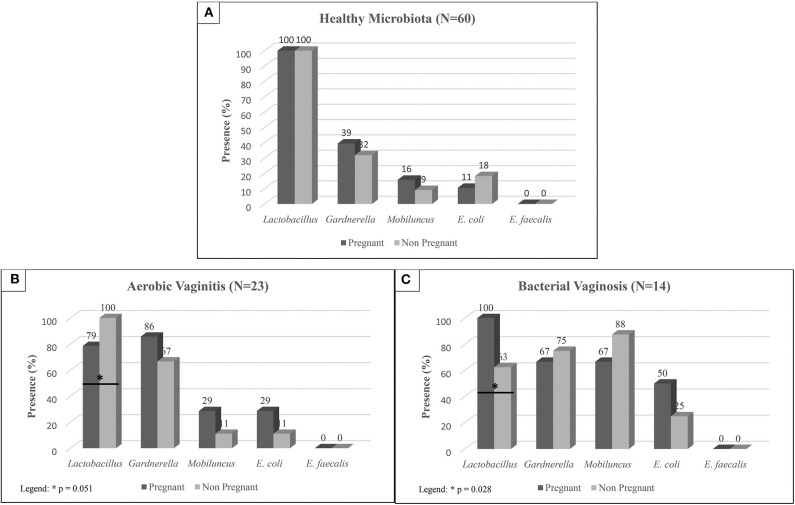
Prevalence of each bacterium in pregnant and non-pregnant women diagnosed as: **(A)** Healthy Microbiota, **(B)** Aerobic Vaginitis, and **(C)** Bacterial Vaginosis according to the microbiological diagnosis. Statistically significant differences were evaluated by Chi-square tests.

Among pregnant and non-pregnant women with healthy microbiota, we found that prevalence of *Lactobacillus* spp. was similar, as shown in Figure 1. On the other hand, pregnant women evidenced higher presence of *Gardnerella* species (39%), and *M. mulieris* (16%) when compared to non-pregnant women (32% *Gardnerella* spp., and 9% of *M. mulieris*).

In the presence of vaginal dysbiosis and infection, no statistically significant differences were found around opportunistic species between pregnant and non-pregnant women, as shown in Figure 1. However, *Lactobacillus* spp. showed statistically significant differences between pregnant and non-pregnant women in both BV and AV cases, as shown in Figure 1. In the case of BV, a higher prevalence of *Lactobacillus* spp. is shown in pregnant women (100%) compared to non-pregnant women (63%; *P* = 0.028). No significant statistically differences were found on the frequency of *Gardnerella* spp. and *M. mulieris* in these samples. In opposition, a drop of *Lactobacillus* spp. prevalence is shown in pregnant women with AV (79%; *P* = 0.051) when compared to non-pregnant women (100%).

### Lactobacilli Quantification by Quantitative Real-Time PCR (qPCR)

Due to the small number of samples with BV, we restricted comparison of the lactobacilli quantification to healthy (60) vs. AV (23) cases.

**Figure 2:**
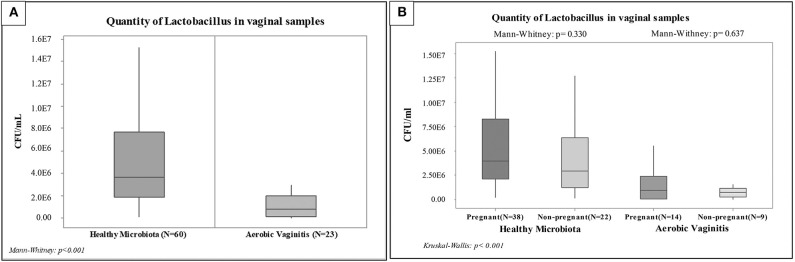
Box plot of the quantification by qPCR of *Lactobacillus* spp. among vaginal samples: **(A)** Non-parametric. Statistical analysis among the overall groups (Healthy Microbiota and Aerobic Vaginitis), **(B)** Non-parametric. Statistical analysis among pregnant and non-pregnant women of each overall group.

Due to the low number of data, a non-parametrical statistical analysis was performed by means of a Mann-Whitney. Significant differences were shown between healthy and AV groups (*P* < 0.001; see Figure 2), whereby *Lactobacillus* spp. varied between 1E6 and 1E7 CFU/ml in healthy microbiota decreased to between 1E3 and 1E5 CFU/ml in AV cases. This was confirmed by Kruskal–Wallis one-way ANOVA testing (*P* < 0.001; see Figure 2).

Mann-Whitney testing indicated no statistically significant differences between pregnant and non-pregnant women with healthy vaginal microbiota and with AV (*P* = 0.330 and *P* = 0.637), as shown in Figure 2. However, the analysis showed statistically significant differences (*P* < 0.001) when comparing pregnant women with healthy microbiota against AV. Likewise, we found slight differences (*P* = 0.006) when comparing non-pregnant women with healthy microbiota against AV. Finally, it is worth noting that the same significance levels were also observed between healthy pregnant women against AV non-pregnant women (*P* < 0.001) and between healthy non-pregnant women against AV pregnant women (*P* = 0.0041). These preliminary results showed similar ranges of lactobacilli load in pregnant and non-pregnant women from each group set (AV and healthy vaginal microbiota).

## Discussion

### Sociodemographic and Behavioral Variables Among Women

This study evaluated a possible relationship between vaginal infection, vaginal dysbiosis and sociodemographic or behavioral variables among pregnant and non-pregnant women. A disruption of the vaginal microbiota usually occurs when any cause promotes a diminution in lactobacilli levels, leading to other microorganisms' augmentation (primary or opportunistic pathogens). These causes of imbalance can be due to several intrinsic and extrinsic factors (Bolton et al., 2008; Borges et al., 2014). As intrinsic factors, the vaginal microbiota of women is driven mainly by hormonal changes during their reproductive life (Farage et al., 2010). These intrinsic factors were distinctively different in the two focus groups (pregnant and non-pregnant women). However, no statistically significant differences regarding composition of vaginal microbiota were detectable (see Table 3). Likewise, in this study, there was no statistically significant relationship among any extrinsic factor (behavioral variables) obtained in the questionnaire by multivariate analysis (see Table 4). This differs from other studies that established statistically significant association with some of the extrinsic factors analyzed by this study, such as the number of sexual partners (Schwebke et al., 1999), and ethnicity (Zhou et al., 2004). Others could not establish associations with the use of contraception, lubricant or spermicide, as well as personal hygiene habits (Keane et al., 1997). Similarly, others did not find any effect of oral contraceptives on the vaginal microbiota of 36 women (Eschenbach et al., 2000). As such, several studies reported contradicting results regarding sociodemographic and behavioral variables, making conclusive comparisons difficult to achieve.

### Prevalence and Types of Vaginal Infection and Vaginal Dysbiosis

In our study set, 52% of women were characterized by a healthy vaginal microbiota, 7% were diagnosed with intermediate vaginal microbiota and 41% with some vaginal infection or vaginal dysbiosis (BV). Similar results were reported in the United Kingdom (Keane et al., 1997), identifying 48% of female participants with healthy vaginal microbiota and 19% with an abnormal microbiota. Similarly, Gondo et al. (2011) reported that 47.5% of the women showed infection in a study enrolling 245 Brazilian women (Gondo et al., 2011). Bacterial vaginosis (BV) is usually reported as the most prevalent vaginal infection around the world (Nelson et al., 2009; Ling et al., 2013; Machado et al., 2013), followed by vulvovaginal candidiasis (VVC) (Ringdahl, 2006). Another condition, aerobic vaginitis (AV), has been recently characterized by Donders and colleagues in 1999, and has been shown to play an important role for vaginal health (Donders, 1999; Datcu et al., 2014; Donders et al., 2017). Furthermore, Donders (2007) showed that this type of vaginal infection could easily be confused with an intermediate microbiota or even bacterial vaginosis (Donders, 2007), which may be a major reason why reliable data on the prevalence of AV in the general population are not very abundant (Donders et al., 2017).

AV can also be associated with the increased risk of preterm pre-labor rupture of membranes, chorioamnionitis, and preterm delivery (Donders et al., 2017). Contrary to previous studies (Schwebke et al., 1996; Cauci et al., 2002; Donders et al., 2005; Vieira-Baptista et al., 2017), in the present study AV was the most prevalent vaginal infection with a similar percentage of AV among pregnant (51.7%) and non-pregnant (48.3%) women. Again, the latter is in contradiction with other studies that reported low AV prevalence among pregnant women. Although Donders et al. (2009) postulated that AV was not common in pregnancy, a more recently publication by Donders et al. (2017) reported that AV could easily be confused with an intermediate microbiota and bacterial vaginosis and so reliable data on the prevalence of AV could be available in few amounts. In 2013, Jahic and colleagues diagnosed AV in 51% of the enrolled female participants, where *E. coli* and *E. faecalis* were the most prevalent bacteria (Jahic et al., 2013). In agreement, Fan et al. (2013) reported the same main bacteria and *S. epidermidis* in their AV cases (Fan et al., 2013).

In non-pregnant women, several studies reported a prevalence of AV between 5 and 10.5% in symptomatic women (Bologno et al., 2011; Marconi et al., 2012; Donders et al., 2017), whereby the most frequently identified bacteria were *E. coli* (4–23%) (Tansarli et al., 2013), *Staphylococcus* (around 27%), *Streptococcus* (0.7–58.7%) and *Enterococcus* spp. (0.3–2.4%) (Von Gruenigen et al., 2000; Iavazzo et al., 2008; Tansarli et al., 2013). These previous studies could partially explain the absence of *E. faecalis* in our study due to the low rate of detection. Finally, *E. coli* prevalence in pregnant (28.57%) and non-pregnant (11.11%), as established in our study, were within the range described by Tansarli et al. (2013) and in agreement with postulations made by Donders (2007).

### Presence of Opportunistic Pathogens in Healthy Microbiota

The vaginal microbiota complexity in healthy and dysbiosis samples had already been described by several authors in women with AV and BV (Tempera and Furneri, 2010; Zozaya-Hinchliffe et al., 2010; Rumyantseva et al., 2016). Similar to Zozaya-Hinchliffe et al. (2010), we believed that the PCR characterization of the major bacterial species by PCR and the development of qPCR assays would be facilitated by first working with specimens whose microbiota would be most likely to differ significantly (Zozaya-Hinchliffe et al., 2010). So, we only selected vaginal samples from sixty women with normal vaginal microbiota who had Nugent scores of 0 and 1, twenty tree women with aggravated AV diagnosis (see section Microbiological Classification of Vaginal Infections), and fourteen women with BV who had Nugent scores of 9 and 10. These 97 women were selected to identify the major bacterial species by PCR, and then healthy and AV women (83 samples) were evaluated by qPCR (see section Amount of *Lactobacillus* spp. Among Healthy Women and Women With Vaginal Infections). However, this selection of samples could be considered a limitation of the present study.

The presence of *Gardnerella* species in a low number in the vaginal microbiota is not an indicator of BV (De Backer et al., 2007; de Vos et al., 2012; Mendling, 2016), being considered as part of the healthy vaginal microbiota. Meanwhile, several studies have shown that there are almost four different groups of *Gardnerella* species (A, B, C, and D), previously all considered as *Gardnerella vaginalis* (Vaneechoutte et al., 2019), which not all of them are related to the development of BV (Santiago et al., 2011; Hardy et al., 2017; Hill et al., 2019). In 2019, Vaneechoutte and colleagues amended several species of *G. vaginalis*, through Matrix-Assisted Laser Desorption/Ionization Time-of-Flight Mass Spectrometry (MALDI-TOF MS), and described then as *Gardnerella leopoldii, Gardnerella piotii* and *Gardnerella swidsinskii*, Therefore, not all *Gardnerella* species detected in several studies constituted *Gardnerella vaginalis* and could explain virulence differences between *Gardnerella* species (Iavazzo et al., 2008; Leite et al., 2010; Muzny and Schwebke, 2013). Since these species could not be delineated using full-length 16S rRNA gene sequences, Hill and colleagues applied partial chaperonin 60 (cpn60) sequences to resolve these four group species (Hill et al., 2019). Both studies showed that *G. swidsinskii* and *G. leopoldii* constituted group A, *G. piotii* corresponded to group B, *G. vaginalis* belonged to group C, and finally, group D was the most diverse subgroup with several *Gardnerella* sp. (such as strains 101, 1500E, 6119V5, and 00703Dmash). However, this last group will require an analysis of additional isolates to establish a species differentiation (Hill et al., 2019; Vaneechoutte et al., 2019). Nonetheless, an abundance of *G. vaginalis* and *G. swidsinskii* was associated with vaginal symptoms of abnormal odor and discharge in their study set (Hill et al., 2019). This heterogeneity and diversity within the genus *Gardnerella* may distinguish clades and how these features may impact BV development (Castro et al., 2020). So, future studies should isolate all *Gardnerella* species of the vaginal samples and further analysis could allow the qPCR methodology to quantify different species of *Gardnerella*.

### Amount of *Lactobacillus* spp. Among Healthy Women and Women With Vaginal Infections

As previously mentioned in results, statistically significant differences were found among the amount of *Lactobacillus* spp. between healthy and AV women (*P* < 0.001). Moreover, lactobacilli load among healthy women was established between 1E6 and 1E7 CFU/ml; meanwhile, the amount of *Lactobacillus* spp. in altered microbiota (AV) was defined between 1E3 and 1E5 CFU/ml. These results are comparable to previous studies with BV (Sha et al., 2005; De Backer et al., 2007; Ling et al., 2011). However, it is important to mention that the specificity of the lactobacilli primers (LactoF: 47.1%%; LactoR: 66.7%) was a limitation of the present study.

### Amount of *Lactobacillus* spp. Among Pregnant and Non-pregnant Women

Furthermore, the results of the present study showed that both healthy and AV pregnant women have a higher concentration of *Lactobacillus* spp. when compared to non-pregnant women of the same categories. These results agree with Walther-António et al. (2014). These authors reported that lactobacilli augmentation during pregnancy and preterm birth help to prevent vaginal infection and counteract higher immune tolerance (Walther-António et al., 2014; Kim et al., 2017). However, there were no statistically significant differences between the amount of *Lactobacillus* spp. of pregnant and non-pregnant women per category. Although the present study is a preliminary analysis of lactobacilli load between pregnant and non-pregnant women, these results point to the possibility to use the same lactobacilli load range to evaluate AV and healthy vaginal microbiota (whether pregnant or non-pregnant) and thus to avoid future vaginal infection establishment in women by monitoring lactobacilli load through qPCR.

These results could corroborate with several studies, which postulated an increment of lactobacilli load in pregnant women (Aagaard et al., 2012; Walther-António et al., 2014). However, there are some major limitations of this study: (1) with 97 participants in PCR assays and 83 participants in qPCR assays, small numbers of particular cases were retained in each subgroup, (2) in PCR assays not all possible species of aerobic bacteria could be targeted in AV samples and, (3) in qPCR assays, normalized concentrations of lactobacilli were realized through low specificity primers for *Lactobacillus* spp. Therefore, future studies must optimize lactobacilli quantification, also quantify certain *Lactobacillus* species and other aerobic bacteria among pregnant and non-pregnant women. Previous studies showed that the presence of different *Lactobacillus* species is a major determinant to the stability of the vaginal microbial community in pregnancy (Verstraelen et al., 2009; Ling et al., 2010). Furthermore, Verstraelen and colleagues demonstrated *L. crispatus* ability to promote and stabilize the normal microbiota while *L. gasseri* and *L. iners* predisposed to some extent to the occurrence of abnormal microbiota (Verstraelen et al., 2009). Future studies should be realized with a bigger and more diverse population set as well as quantification of specific *Lactobacillus* species (such as *L. crispatus, L. gasseri*, and *L. iners*) as postulated by others authors (Verstraelen et al., 2009; Walther-António et al., 2014; Vaneechoutte, 2017a).

## Data Availability Statement

All datasets generated for this study are included in the article/Supplementary Material.

## Ethics Statement

This study was approved by the Ethics Committee of Universidad San Francisco de Quito (USFQ) and the Ministry of Health of Ecuador (Protocol code: 2016-140M by MSP-SDM-10-2013-2019-O review board). The volunteers were recruited to our study set, after having read and signed the informed consent or, in the case of the underage volunteers, from their parents or legal representatives.

## Author Contributions

AM, GV, and DP-H were responsible for modeling and experimental design. Samples collection oversaw doctors of Gynecology and Obstetrics Service of Carlos Andrade Marin Hospital (HCAM), Gynecological-Obstetric Hospital Isidro Ayora (HGOIA), and Center for Teaching Health Cipriana Dueñas. The DNA extraction was performed at Hospital Carlos Andrade Marín by CC-B. Molecular characterization was conducted by DP-H in the Microbiology Institute at USFQ. Biostatistics analysis was developed by VB and DP-H at USFQ. All authors contributed to the article and approved the submitted version.

## Conflict of Interest

The authors declare that the research was conducted in the absence of any commercial or financial relationships that could be construed as a potential conflict of interest.
