# Supplementary figures and images for: Vaginal Microbiota Evaluation and Lactobacilli Quantification by qPCR in Pregnant and Non-pregnant Women: A Pilot Study
Source: Front Cell Infect Microbiol. 2020 Jun 19;10:303. doi: 10.3389/fcimb.2020.00303 (PMC7318849; doi:10.3389/fcimb.2020.00303)

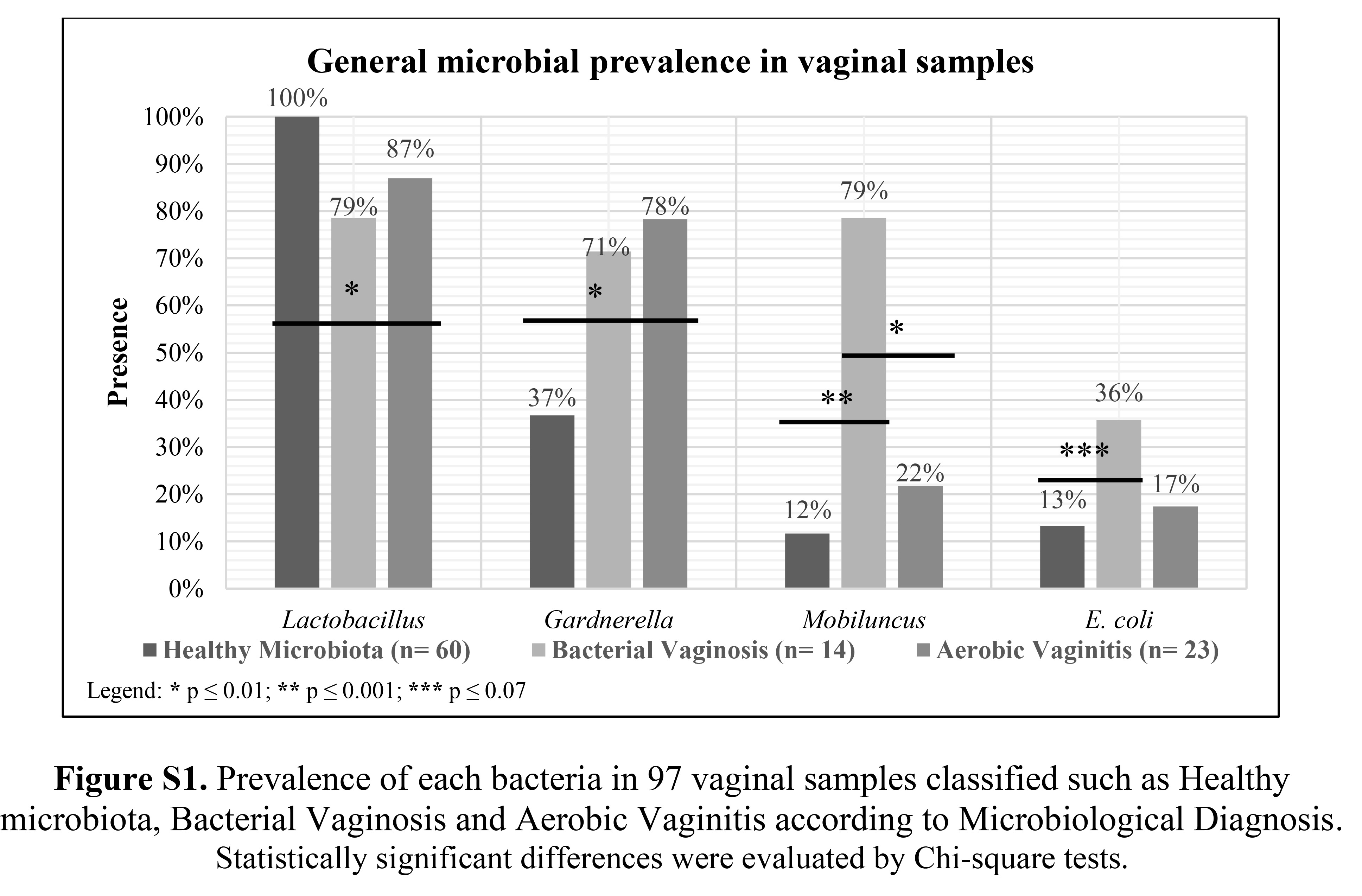

Supplement: Supplementary file 1 [file Image_1.jpeg]
